# Edoxaban Dosing Time Affects Blood Coagulation Inhibition in Rats

**DOI:** 10.1055/s-0041-1725041

**Published:** 2021-04-14

**Authors:** Naoto Nagata, Muneo Kawasumi, Akio Fujimura, Hitoshi Ando

**Affiliations:** 1 Department of Cellular and Molecular Function Analysis, Kanazawa University Graduate School of Medical Science, Kanazawa, Japan; 2 Department of Pharmacology, Jichi Medical University, Shimotsuke, Japan

**Keywords:** blood coagulation, circadian rhythm, chronotherapy, edoxaban, pharmacokinetics

## Abstract

Coagulation–fibrinolytic system activity shows daily rhythmicity, with hypercoagulability in the morning and hypocoagulability in the evening. Consequently, the efficacy of anticoagulants may be influenced by their dosing time. Edoxaban, a selective inhibitor of the active form of coagulation factor X (FXa), is taken orally once daily, but the optimal dosing time is unknown. This study evaluated the dosing time-dependent effects of edoxaban on coagulation activity and thrombus formation in rats. Edoxaban (10 mg/kg) or vehicle was administered to Wistar rats at zeitgeber time (ZT)-2 (beginning of the light phase) or ZT14 (beginning of the dark phase), followed by blood collection at ZT4, ZT10, ZT16, or ZT22, to measure the activity of coagulation factors and edoxaban concentrations, or followed by inferior vena cava ligations at ZT4 or ZT16, to assess the efficacy of edoxaban against thrombus formation. Coagulation FX activity was high during the light phase, and a single dose of edoxaban administered at ZT2 inhibited FX activity and thrombus formation more potently compared with the same dose administered at ZT14. The inhibitory effects during the light phase could be attributed, at least in part, to the high blood concentration of edoxaban achieved by dosing at ZT2. Morning dosing of edoxaban leads to a high blood concentration of the drug during the morning hours and thus may better counteract the hypercoagulability and hypofibrinolytic activity characteristic of the morning hours. Optimizing the dosing time may contribute to improving the efficacy of edoxaban.

## Introduction

The activity of the coagulation–fibrinolytic system follows a daily rhythm, 1 2 such that the thrombotic tendency is higher in the morning and lower in the evening. Consequently, the occurrence of acute myocardial infarction 3 4 and ischemic stroke 5 6 tends to peak during the morning hours. A circadian rhythmicity also marks the activity of blood coagulation factors in clinically healthy individuals, with factor (F) VII values peaking between 8 a.m. and noon 7 and FVIII and FIX values peaking at approximately 9 a.m. 8 9 The fibrinolytic activity of tissue-type plasminogen activator also exhibits a daily rhythmicity, peaking at approximately 6 p.m., whereas the activity of its inhibitor, plasminogen activator inhibitor-1, is the highest in the early morning. 2 10 Tissue factor pathway inhibitor, an activated FX (FXa)-dependent direct inhibitor of the activated tissue factor/FVII complex, also follows a daily pattern throughout the day. 11 In accordance with the rhythmicity of these components of the coagulation–fibrinolytic system, (1) the prothrombin time (PT) and activated partial thromboplastin time (APTT) are lowest during the morning, 9 and (2) the effect of anticoagulant/fibrinolytic drugs may depend on dosing time. In fact, the anticoagulant effect on the anti-FXa activity of unfractionated heparin infusions was shown to increase at night but to decrease markedly in the morning. 12 13 Dosing time-dependent effects of fibrinolytic therapy, with attenuation in the morning, have also been reported. 13

Recently, direct oral anticoagulants (DOACs) have emerged as an alternative to vitamin-K antagonists such as warfarin. DOACs are as effective as warfarin in preventing stroke in patients with nonvalvular atrial fibrillation and in the treatment of venous thromboembolism. 14 15 16 17 The clinical advantages of DOACs over conventional anticoagulants include less frequent and less severe bleeding, oral availability, a rapid onset of action, fixed dosing, minimal drug and dietary interactions, and no need for routine coagulation monitoring. 14 15 16 17 Moreover, in contrast to vitamin-K antagonists, DOACs inhibit only one component of the coagulation cascade. For example, apixaban, rivaroxaban, and edoxaban inhibit FXa, whereas dabigatran inhibits thrombin (activated FII [FIIa]). FX is activated by both the intrinsic and extrinsic coagulation pathways, and FXa subsequently interacts with FVa to convert prothrombin into thrombin.

Edoxaban, a direct, selective, and reversible inhibitor of FXa, is the newest DOAC and is taken once daily. 18 Edoxaban, like other DOACs, is absorbed rapidly when taken orally and has a relatively short-elimination half-life. 19 In healthy individuals, the time to peak blood concentration and the terminal elimination half-life of edoxaban following a single dose of 30 to 150 mg are 1 to 2 and 8 to 11 hours, respectively, and the accumulation after daily dosing is approximately 1.1. 20 Thus, edoxaban does not accumulate to a clinically significant level even after multiple doses. 20 Given the daily rhythmicity of coagulation–fibrinolytic system activity and the pharmacokinetics of edoxaban, we hypothesized that the dosing time of this DOAC determines its anticoagulant effect. Thus, in the present study, we evaluated the dosing time-dependent effects of edoxaban on coagulation activity and thrombus formation in Wistar rats.

## Methods

### Animals

Male Wistar rats (Japan SLC, Hamamatsu, Japan) were obtained at 8 weeks of age and maintained on a 12-hour light/12-hour dark cycle (lights on between 8:45 a.m. and 8:45 p.m.) at 24 to 26°C with free access to water and a regular diet (CRF-1; Charles River Laboratories Japan, Yokohama, Japan). The animals were used for the experiments at 10 weeks of age. All animal procedures were performed in accordance with the Guidelines for the Care and Use of Laboratory Animals of Kanazawa University, and all animal protocols were approved by the Institutional Animal Care and Use Committee of Kanazawa University (protocol number: AP-173838).

### Experiment 1: Diurnal Variation in the Activity of Coagulation Factors

The rats were anesthetized with pentobarbital (30 mg/kg, i.p.). Blood samples were collected in 3.2% sodium citrate tubes (Terumo Corporation, Tokyo, Japan) by cardiac puncture at the following zeitgeber times (ZTs): 0, 4, 8, 12, 16, and 20 hours, with ZT0 defined as lights on and ZT12 as lights off (8:45 a.m. and 8:45 p.m., respectively). The plasma samples were centrifuged (1,500 g) at 4°C for 10 minutes and then stored at −80°C until assayed. The plasma activities of FII, FV, and FX were determined by measuring the clotting time of a sample using the appropriate reagents (factor-deficient plasma and the HemosIL RecombiPlasTin kit; IL Japan, Tokyo, Japan) and an automated coagulation analyzer (ACL TOP; IL Japan). The activity levels were expressed as percentages (relative to normal human plasma). PT and APTT were measured using a coagulation analyzer (CA-510; SYSMEX Corporation, Kobe, Japan) according to the manufacturer's instructions.

### Experiment 2: Dosing Time-Dependent Effects of Edoxaban on Coagulation Activity

The rats were divided into four groups and orally administered edoxaban (10 mg/kg; Daiichi Sankyo Co., Ltd, Tokyo, Japan) dissolved in 0.5% (w/v) methyl cellulose (Wako Pure Chemical, Osaka, Japan) or administered vehicle only (0.5% methyl cellulose) at ZT2 or ZT14. The dosage of edoxaban was selected based on previous studies. 21 22 At 2, 8, 14, and 20 hours after dosing (i.e., ZT4, ZT10, ZT16, and ZT22 for dosing at ZT2; ZT16, ZT22, ZT4, and ZT10 for dosing at ZT14), the rats were anesthetized with pentobarbital (30 mg/kg, i.p.), and blood samples were collected in 3.2% sodium citrate tubes and sodium heparin tubes (Terumo Corporation) for coagulation activity assays and edoxaban plasma concentration measurements, respectively. The plasma concentrations of edoxaban were measured by high-performance liquid chromatography with tandem mass spectrometry, as described elsewhere. 18

### Experiment 3: Dosing Time-Dependent Effects of Edoxaban on Thrombus Formation

The rats were orally administered edoxaban (10 mg/kg) or vehicle at ZT2 or ZT14. At 2 or 14 hours after dosing (i.e., ZT4 or ZT16), venous thrombus formation was monitored according to Hladovec's method. 23 Briefly, rats anesthetized with pentobarbital received an injection of hypotonic saline (0.225%) into the femoral vein (5 mL/kg/min for 2 minutes), and the inferior vena cava was then ligated using 4–0 silk suture just below the left renal vein. Ten minutes later, the vena cava was ligated again, 1.5 cm below the first ligation. The thrombus that formed between the ligation sites was collected 90 minutes after the second ligation. The protein content of the thrombus was measured using a bicinchoninic acid assay kit (Thermo Fisher Scientific, Waltham, Massachusetts, United States).

### Statistical Analyses

The overall difference between more than two groups was determined using one-way analysis of variance (ANOVA), followed by Tukey's post hoc test to determine the differences between individual groups. All calculations were performed using SPSS Statistics (v24.0, IBM, Armonk, New York, United States). The data are expressed as the mean ± SD. A *p* -value of <0.05 was considered to indicate statistical significance. The data were plotted graphically using GraphPad Prism version 8.4.3 for Windows (GraphPad Software, San Diego, California, United States).

## Results

### Diurnal Variation in the Activity of Coagulation Factors

**Fig. 1:**
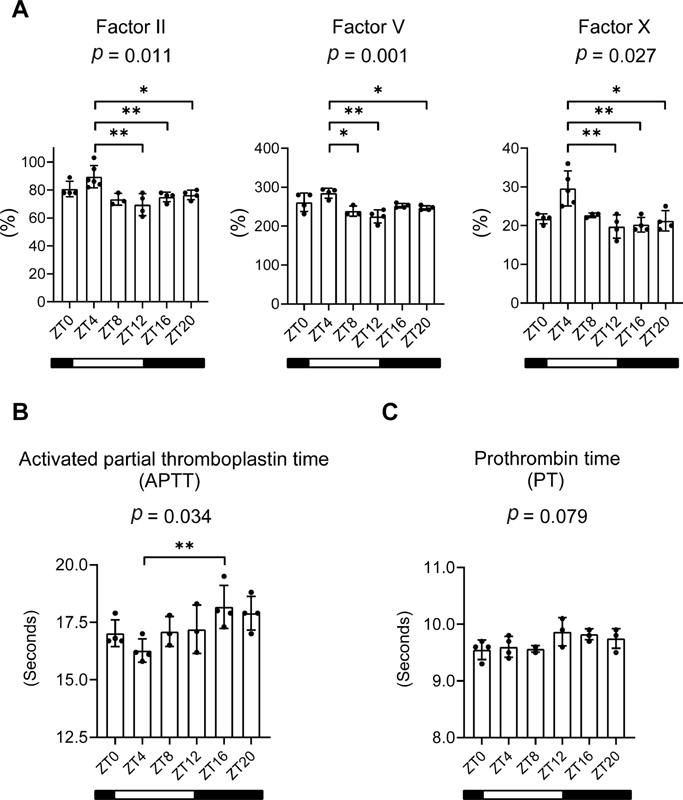
Diurnal variations in coagulation factor activities in rats. Blood samples were obtained at zeitgeber times (ZT) 0, 4, 8, 12, 16, and 20. ( **A** ) The plasma activities of factors II, V, and X. ( **B** ) Activated partial thromboplastin time. ( **C** ) Prothrombin time. White and black horizontal bars indicate the light and dark periods, respectively. Data represent the means ± standard deviation ( *n* = 3–6). The *p* -value (analysis of variance) is presented at the top of each panel. * *p* < 0.05, ** *p* < 0.01 versus ZT4.

Consistent with the previous study, 24 the plasma activities of FII, FV, and FX exhibited diurnal variation, with a peak in the middle of the light phase and a trough at the beginning of the dark phase ( Fig. 1A ). In parallel, the APTT was significantly longer at ZT16 than at ZT4 ( Fig. 1B ). The diurnal variation in PT followed a similar trend, but the difference between the two ZTs did not reach statistical significance ( Fig. 1C ).

### Dosing Time-Dependent Effects of Edoxaban on Coagulation Activity

**Fig. 2:**
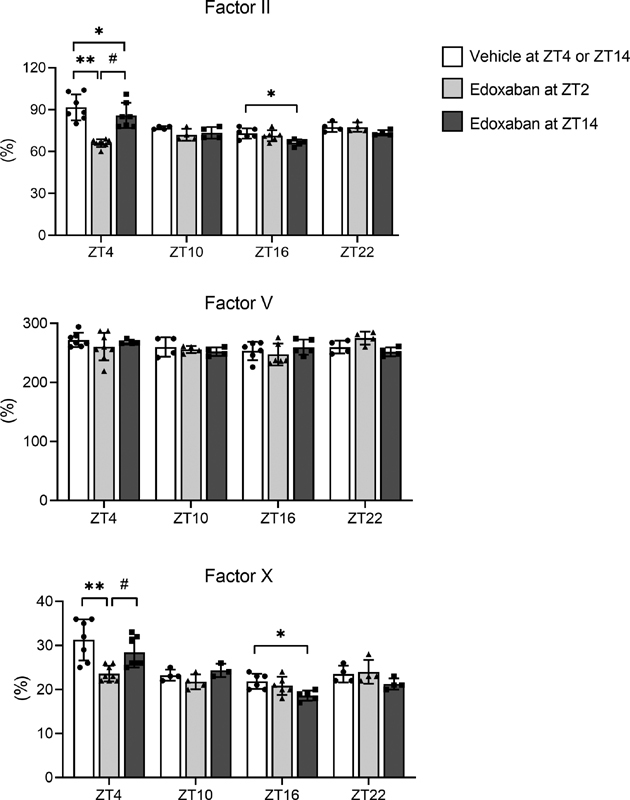
Dosing time-dependent effects of edoxaban on coagulation factor activity. Rats were administered vehicle solution or edoxaban (10 mg/kg) at zeitgeber time (ZT)-2 (light gray bars) or ZT14 (dark gray bars). Blood samples were obtained at 2, 8, 14, and 20 hours after dosing (i.e., at ZT4, ZT10, ZT16, or ZT22 for dosing at ZT2; at ZT16, ZT22, ZT4, or ZT10 for dosing at ZT14). The plasma activities of factors II, V, and X were compared at each time point. The combined data from vehicle-administered rats at ZT2 or ZT14 are shown (white bars). The data represent the mean ± standard deviation ( *n* = 4–8). * *p* < 0.05, ** *p* < 0.01 versus that of the vehicle-administered group at the same ZT. ^#^ *p* < 0.05 versus edoxaban dosing in the ZT2 group at the same ZT.

The potential dosing time-dependent effects of edoxaban on coagulation factor activity were examined using ZT2 and ZT14 as the dosing time points, followed by comparisons with the coagulation factor activities at 2, 8, 14, and 20 hours. Compared with vehicle administration or edoxaban dosing at ZT14, edoxaban dosing at ZT2 significantly lowered FII and FX activities at ZT4 (2 hours after dosing; Fig. 2 ). Compared with vehicle administration, edoxaban dosing at ZT14 significantly decreased FII and FX activities at ZT16 (2 hours after dosing) and significantly decreased FII activity at ZT4 (14 hours after dosing; Fig. 2 ). At ZT16, there are no significant differences in FII and FX activities between the two groups of edoxaban dosing. The activity of FV was not influenced by the administration of edoxaban at the either dosing time points ( Fig. 2 ). Together, the inhibitory effects of edoxaban on FII and FX activities during the light phase were greater after dosing at ZT2 than after dosing at ZT14.

### Dosing Time-Dependent Effects of Edoxaban on Thrombus Formation

**Fig. 3:**
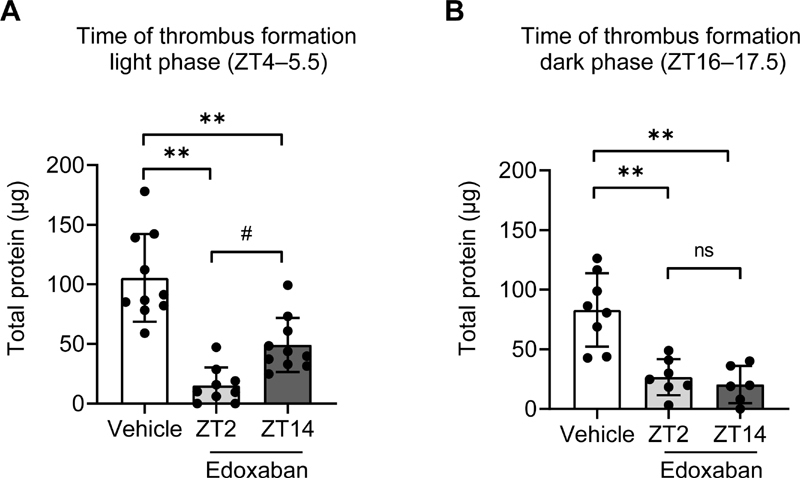
Dosing time-dependent effects of edoxaban on thrombus formation. Rats were administered vehicle (white bars) or edoxaban (10 mg/kg) at zeitgeber time (ZT)-2 (light gray bars) or ZT14 (dark gray bars). Venous thrombosis formation was initiated at ( **A** ) 2 or ( **B** ) 14 hours after dosing (i.e., at ZT4 or ZT16). The combined data from vehicle-administered rats at ZT2 or ZT14 are presented (white bars). Data represent the means ± standard deviation ( **A** , *n* = 9–10; **B** , *n* = 6–8). ** *p* < 0.01 versus the vehicle-administered group. ^#^ *p* < 0.05 versus edoxaban dosing in the ZT2 group. ns, not significant.

Additionally, we investigated the influence of the dosing time of edoxaban (dosing at the light phase [ZT2] or dark phase [ZT14]) on the inhibition of venous thrombus formation. During the light phase (ZT4–5.5), the both timing of edoxaban dosing significantly inhibited thrombus formation compared with vehicle administration ( Fig. 3A ). Intriguingly, edoxaban dosing at ZT2 inhibited thrombus formation more potently than did edoxaban dosing at ZT14 ( Fig. 3A ). Whereas, during the dark phase (ZT16–17.5), inhibitory effects of edoxaban on thrombus formation were comparable between the two ZTs ( Fig. 3B ).

### Effect of Dosing Time on the Pharmacokinetics of Edoxaban

**Fig. 4:**
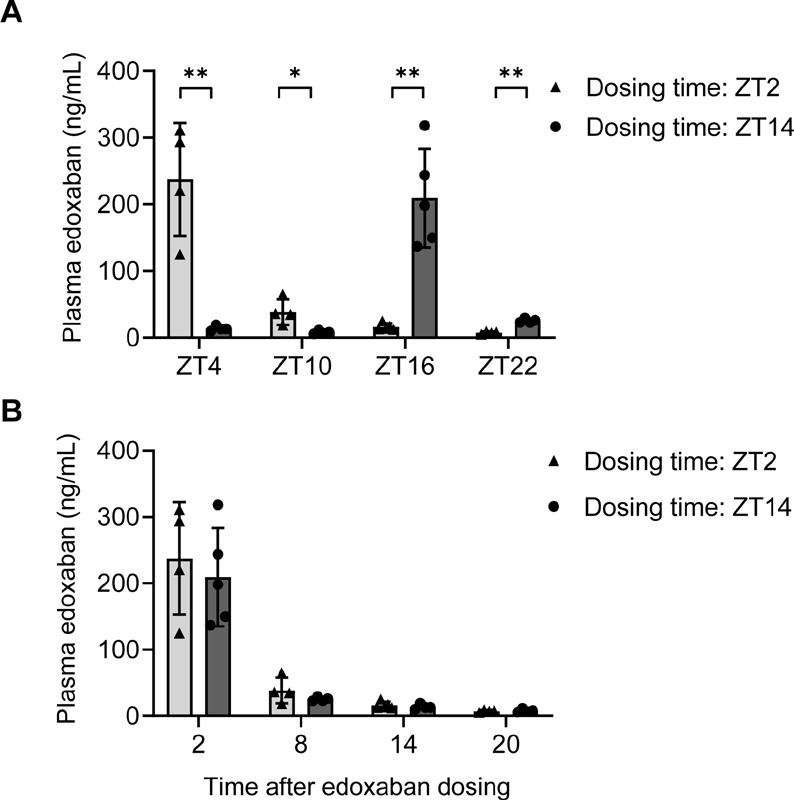
Effect of dosing time on the pharmacokinetics of edoxaban. Rats were administered edoxaban (10 mg/kg) at zeitgeber time (ZT)-2 (light gray bars) or ZT14 (dark gray bars). Blood samples were obtained at 2, 8, 14, and 20 hours after dosing. The plasma edoxaban concentrations ( **A** ) at each ZT and ( **B** ) at the indicated times after dosing. Data represent the means ± standard deviation ( *n* = 4–5). * *p* < 0.05, ** *p* < 0.01 versus edoxaban dosing of the ZT2 group at the same ZT.

The correlation between the anticoagulant activity and plasma concentration of edoxaban 20 is indicative of the drug's rapid onset of action. In the present study, at ZT4 and ZT10, the plasma concentrations of edoxaban were significantly higher after dosing at ZT2 than at ZT14 ( Fig. 4A ). Conversely, at ZT16 and ZT20, plasma edoxaban concentrations were significantly higher after dosing at ZT14 than at ZT2 ( Fig. 4A ). Although the dosing time affects the pharmacokinetics of many drugs, the plasma concentrations of edoxaban after ZT2 and ZT14 dosing were comparable until 20 hours after dosing ( Fig. 4B ). Therefore, the dosing time-dependent effects of edoxaban on plasma FX activity ( Fig. 2 ) and thrombus formation during the light phase ( Fig. 3A ) may, at least in part, be due to differences in the plasma concentrations of edoxaban at the different dosing times.

## Discussion

The approved dosage of edoxaban for adults is 30 to 60 mg orally once a day, but the optimal dosing timing is unclear. Our study showed that FX has high activity during the light phase, and that a single dose of edoxaban administered at the beginning of the light phase (ZT2) had stronger inhibitory effects on FX activity and thrombus formation compared with the same dose administered at the beginning of the dark phase (ZT14). These findings suggest that morning administration of edoxaban leads to a high blood concentration during the morning hours, which would more effectively counteract the hypercoagulability and hypofibrinolytic state characteristic of this time of day. Clinical studies are needed to translate this observation into clinical benefit.

The daily rhythm of total coagulability is similar between humans 1 and rats. 25 In our study, the circadian variation in total coagulability was reflected by higher FII, FV, and FX activities in the animals during the light than the dark phases ( Fig. 1A ). For FII and FX, their circadian variation was matched by the dosing time-dependent inhibitory effects of edoxaban on coagulation factors' activities and on thrombus formation during the light phase. The chronopharmacology of edoxaban can be attributed, at least in part, to the daily rhythm of the coagulability and to differences in the plasma concentrations of edoxaban between dosing times. Intriguingly, during the dark phase, edoxaban dosing at ZT2 inhibited thrombus formation to a similar extent as did edoxaban dosing at ZT14 ( Fig. 3B ), although the plasma concentrations of edoxaban were relatively low (∼15 ng/mL) at 14 hours after dosing ( Fig. 4 ). This discrepancy can be partly explained by the lower coagulability that occurs during the dark phase than during the morning, but further investigations are needed.

## Limitations

Whether our results are directly applicable to humans remains to be determined due to some research limitations. First, despite the similarity in the daily rhythm of total coagulability between humans and rats, the coagulation factors differ somewhat. Thus, while FII and FVII activities exhibit circadian variations in humans and rats, circadian variation in FX activity has been reported only in rats. 24 25 Second, the present study did not consider the diurnal variation of the fibrinolytic activity. Third, the effect of multiple doses of edoxaban remains unclear. Although the drug does not accumulate after multiple doses, 20 whether multiple doses nonetheless alter the 24-hour rhythm of the coagulation–fibrinolytic system is yet unknown. Finally, the impact of the dosing time on the pharmacokinetics of edoxaban in humans remains unclear. Future clinical studies are needed to determine whether the impact of the edoxaban dosing time on its pharmacokinetics is clinically significant. In healthy individual, edoxaban has high oral bioavailability (62%). 26 Renal clearance of the drug accounts for approximately 40% of its total clearance, with metabolism and biliary secretion accounting for the remaining 60%. 27 Unchanged edoxaban is the most abundant species in urine (∼25% of the dose) and feces (∼50%), with modest metabolism (<25% of the dose) occurring primarily by hydrolysis 27 and minor contributions (<4% of the total dose) by metabolites M-6 and M-8, which are formed via CYP3A activity. 27 These drug metabolic profiles suggest that circadian variations in the activities of CYP enzymes have minimal effects on the metabolism of edoxaban, consistent with our results in rats ( Fig. 4B ). For several cytochrome-P450 (CYP)-dependent drugs, including rivaroxaban, another once-daily DOAC, considerable variation in their pharmacokinetics according to dosing time has been related to circadian variation in the activity of the enzymes responsible for their metabolism. 28 29 30 Notably, Brunner-Ziegler et al reported that evening dosing of rivaroxaban leads to prolonged exposure to rivaroxaban concentrations and better matches the morning hypofibrinolysis, 28 in contrast to edoxaban of this study.

## Conclusion

In conclusion, this study showed that the dosing time of edoxaban can influence its anticoagulant effects during the light phase, when blood coagulability is high. Thus, optimization of the dosing time of edoxaban may contribute to improve its efficacy.
